# Nb/N Co-Doped Layered Perovskite Sr_2_TiO_4_: Preparation and Enhanced Photocatalytic Degradation Tetracycline under Visible Light

**DOI:** 10.3390/ijms231810927

**Published:** 2022-09-18

**Authors:** Jiansheng Wang, Pengwei Li, Yingna Zhao, Xiongfeng Zeng

**Affiliations:** 1 College of Materials Science and Engineering, North China University of Science and Technology, Tangshan 063210, China; 2 Hebei Province Laboratory of Inorganic Nonmetallic Materials, North China University of Science and Technology, Tangshan 063210, China; 3 Hebei (Tangshan) Ceramic Industry Technology Research Institute, Tangshan 063007, China

**Keywords:** layered perovskite, Sr_2_TiO_4_, Nb/N co-doping, tetracycline, photocatalyst

## Abstract

Sr_2_TiO_4_ is a promising photocatalyst for antibiotic degradation in wastewater. The photocatalytic performance of pristine Sr_2_TiO_4_ is limited to its wide bandgap, especially under visible light. Doping is an effective strategy to enhance photocatalytic performance. In this work, Nb/N co-doped layered perovskite Sr_2_TiO_4_ (Sr_2_TiO_4_:N,Nb) with varying percentages (0–5 at%) of Nb were synthesized by sol-gel and calcination. Nb/N co-doping slightly expanded the unit cell of Sr_2_TiO_4_. Their photocatalytic performance towards antibiotic (tetracycline) was studied under visible light (λ > 420 nm). When Nb/(Nb + Ti) was 2 at%, Sr_2_TiO_4_:N,Nb(2%) shows optimal photocatalytic performance with the 99% degradation after 60 min visible light irradiation, which is higher than pristine Sr_2_TiO_4_ (40%). The enhancement in photocatalytic performance is attributed to improving light absorption, and photo-generated charges separation derived from Nb/N co-doping. Sr_2_TiO_4_:N,Nb(2%) shows good stability after five cycles photocatalytic degradation reaction. The capture experiments confirm that superoxide radical is the leading active species during the photocatalytic degradation process. Therefore, the Nb/N co-doping in this work could be used as an efficient strategy for perovskite-type semiconductor to realize visible light driving for wastewater treatment.

## 1. Introduction

Tetracycline (TC) is a widely used broad-spectrum antibiotic in medical treatment, animal husbandry, and aquaculture [1]. The accumulation of antibiotics in the environment leads to drug-resistant bacteria, which could bring serious threats to the ecological environment and human health [2,3,4,5]. Tetracycline is not only hard to self-degrade in the natural environment [3], but also difficult to be eliminated by conventional techniques.

Photocatalytic technology [6,7,8,9] utilizes a photocatalyst to generate light-generated holes and electrons under irradiation. Holes or electrons react with H_2_O or O_2_ to generate superoxide radicals and hydroxyl radicals with strong oxidation capacity, thereby thoroughly oxidizing and degrading organic pollutants [10] in wastewater. The core of photocatalytic oxidation technology are photocatalysts. Among many photocatalytic materials, strontium titanate has attracted wide attention because of its advantages, such as being inexpensive, pollution-free, and light corrosion resistant [11]. However, strontium titanate also has the disadvantages of having a large bandgap and high photo-generated carrier recombination rate. Layered perovskite Sr_2_TiO_4_ exhibits higher photocatalytic performance than perovskite SrTiO_3_, due to the particularly layered crystal structure and typical 2D charge transportation properties [12,13].

The wide bandgap of Sr_2_TiO_4_ exhibits photocatalytic activity only under ultraviolet light, which gravely affects further enhancement of photocatalytic performance. Ion doping can reduce the bandgap [14] of the perovskite material [11], including single metal doping, nonmetal doping, and co-doping. For instance, by introducing Cr [15], Ag [16], F [17], chalcogens [18], La/N [13], Cr/F [19], La/Rh [20], and La/Fe [12], the light response range of Sr_2_TiO_4_ is extended. Properties of Sr_2_TiO_4_ doping with different ions was shown in Table 1. Among them, co-doping has gained more interest, because co-doping is expected to maintain the charge-balance without forming oxygen vacancies.

Among various doping elements for oxide semiconductors, doping the nitrogen at the O site can narrow the band gap through the hybridization N 2p with the O 2p [21]. Meanwhile, N-doped oxide semiconductors are often accompanied by oxygen vacancies [22] due to charge compensation. Too high of a concentration of oxygen vacancies will act as a charge carrier recombination center, which is detrimental to photocatalytic activity [16,23,24]. The preparation methods of Sr_2_TiO_4_ mainly includes conventional solid-state reactions, the molten-salt method, and sol-gel method. Among them, the sol-gel synthesis process can achieve molecular-level doping.

This study chooses N-doped Sr_2_TiO_4_, where N^3−^ replaces O^2−^ to regulate the band structure. In order to control the concentration of oxygen vacancies, we replace Ti^4+^ (r = 0.061 nm) ions with Nb^5+^ (r = 0.064 nm) to balance the negative charge introduced by the unequal substitution of N^3−^ and O^2−^ Nb/N co-doped Sr_2_TiO_4_, which perhaps obtains charge-balanced Sr_2_TiO_4_:N,Nb and avoids too high of a concentration of oxygen vacancies. The amount of the dopant should also be optimized. Hence, we perform an investigation on Nb/N co-doped layered perovskite Sr_2_TiO_4_ for photocatalytic degradation tetracycline by varying Nb^5+^ doping content. To the authors’ knowledge, although there have been several studies on the doping modification of Sr_2_TiO_4_ [12,13,15,16,17,19,20] for photocatalytic hydrogen production, there is no literature report on the preparation and photocatalytic degradation performance of Nb/N co-doped Sr_2_TiO_4_. In this work, Sr_2_TiO_4_:N,Nb(2%) shows the best photocatalytic degradation performance towards tetracycline under visible light. The superior photocatalytic performance can be attributed to N-doping, narrowing the bandgap and Nb-doping compensating charge imbalance. This work affords a new insight to realizing visible-light-driven perovskite-type semiconductors.

## 2. Results and Discussion

### 2.1. Materials Characterization

**Figure 1:**
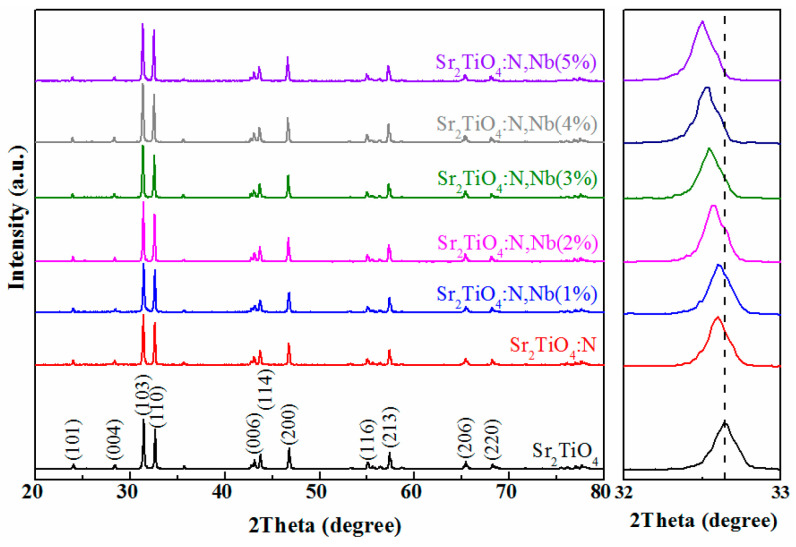
XRD patterns and partial enlarged view of Sr_2_TiO_4_, Sr_2_TiO_4_:N, and Sr_2_TiO_4_:N,Nb.

As shown in Figure 1, the crystal structure of Sr_2_TiO_4_, Sr_2_TiO_4_:N and Sr_2_TiO_4_:N,Nb with different Nb-doping content were investigated by X-ray diffraction (XRD). There was only one phase of Sr_2_TiO_4_ (JCPDS NO:39-1471) without preferred growth orientation in the XRD patterns. Characteristic peaks of 2*θ* at 23.9, 28.3, 31.4, 32.6, 43.0, 43.7, 46.7, 55.0, 57.3, 65.4 and 68.2° were indexed to (101), (004), (103), (110), (006), (114), (200), (116), (213), (206) and (220) planes of Sr_2_TiO_4_ (JCPDS NO:39-1471), respectively.

After N-doping, the peak of (110) shown by Sr_2_TiO_4_:N in XRD shifted to a lower angle, meaning larger d-spacing than that of Sr_2_TiO_4_, and this larger spacing proved that N-doped Sr_2_TiO_4_ successfully. This is due to the N^3−^ radius (0.146 nm) being slightly larger than the O^2−^ radius (0.140 nm); when N^3−^ enters the Sr_2_TiO_4_ crystal lattice, the crystal lattice expands. According to Bragg’s equation 2*d*sin*θ* = *λ*, with the increase of *d*-spacing, the diffraction angle 2*θ* shifts to a lower angle. Similar to Sr_2_TiO_4_:N, 2*θ* of Sr_2_TiO_4_:N,N shifted to a lower direction gradually with the increase of Nb-doping content, as shown in Figure 1. Because the Nb^5+^ radius (0.064 nm) and N^3−^ radius (0.146 nm) are slightly larger than the Ti^4+^ radius (0.061 nm) and O^2−^ radius (0.140 nm), respectively, the more the Nb-doping content, the more the lattice expansion [25,26], and the more diffraction angle offset. According to the XRD patterns, it can be concluded that both Nb^5+^ and N^3−^ were doped into the lattice of Sr_2_TiO_4_ successfully. The average crystallite sizes were calculated by Scherrer’s equation [14] of the Sr_2_TiO_4_ (103) XRD reflection and were shown in Table 2.

**Figure 2:**
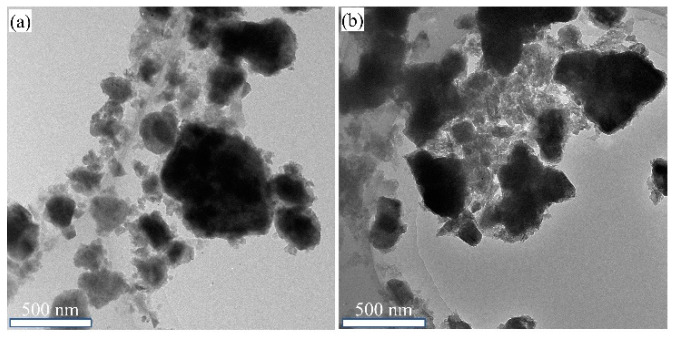
TEM images of (**a**) Sr_2_TiO_4_; (**b**) Sr_2_TiO_4_:N,Nb(2%).

The morphological characterization of Sr_2_TiO_4_ and Sr_2_TiO_4_:N,Nb(2%) were shown in Figure 2. After being calcined at 1000 °C for 4 h, both pristine Sr_2_TiO_4_ and Sr_2_TiO_4_:N,Nb(2%) were composed of irregular particles ranging from tens of nanometers to 1 micron.

**Figure 3:**
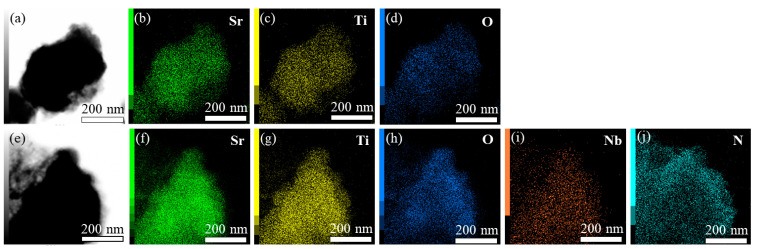
(**a**) TEM image of Sr_2_TiO_4_; (**b**–**d**) Elemental mapping of Sr, Ti, and O; (**e**) TEM image of Sr_2_TiO_4_:N,Nb(2%); (**f**–**j**) Elemental mapping of Sr, Ti, O, Nb, and N.

TEM elemental mapping of Sr_2_TiO_4_ was performed and the results were shown in Figure 3a–d. It verified the existence of Sr, Ti, O, and these elements were distributed evenly. TEM elemental mapping of Sr_2_TiO_4_:N,Nb(2%) was also performed and the results were shown in Figure 3f–j. It confirmed the existence of Sr, Ti, O, Nb, and N, and these elements were evenly distributed as well.

**Figure 4:**
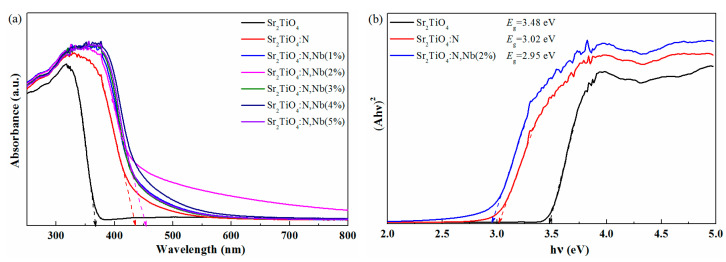
UV-Vis spectra (**a**) and Tauc plots (**b**) of Sr_2_TiO_4_, Sr_2_TiO_4_:N, and Sr_2_TiO_4_:N,Nb(2%).

Figure 4a shows the absorbance of Sr_2_TiO_4_, Sr_2_TiO_4_:N, and Sr_2_TiO_4_:N,Nb. The UV-Vis absorption spectra of the pristine and doped Sr_2_TiO_4_ showed a significant difference. The absorption edge of pristine Sr_2_TiO_4_ is located in the ultraviolet region. However, the absorption edge of N doping and Nb/N co-doping Sr_2_TiO_4_ were significantly red and shifted into the visible light region, and Sr_2_TiO_4_:N,Nb(2%) showed the strongest absorption capacity of visible light. According to the previous literature, N doping can reduce the band gap through hybridization of N 2p with the O 2p, raising the valence band maximum. The energy level of Nb^5+^ is similar to titanium 3d orbital energy, so Nb can replace Ti and mix Nb 4d with Ti 3d orbitals without lowering the conduction band minimum [27]. The same observation has been seen in other metal oxides doped with nitrogen, such as N-doped SrTiO_3_ [28], N-doped NaLaTiO_4_ [29], N-doped Sr_2_TiO_4_ [29], Nb/N co-doped TiO_2_ [27], and so on. The bandgap *E*_g_ deduced from the Tauc plots in Figure 4b were 3.48 eV, 3.02 eV, and 2.95 eV for pristine Sr_2_TiO_4_, Sr_2_TiO_4_:N, and Sr_2_TiO_4_:N,Nb(2%), respectively. Pristine Sr_2_TiO_4_ had a wide bandgap (3.48 eV), which was consistent with the previous literature [13]. N doping and Nb/N co-doping can reduce the bandgap of Sr_2_TiO_4_. Similar phenomena have also been observed in other materials involving N doping [13,24,30].

**Figure 5:**
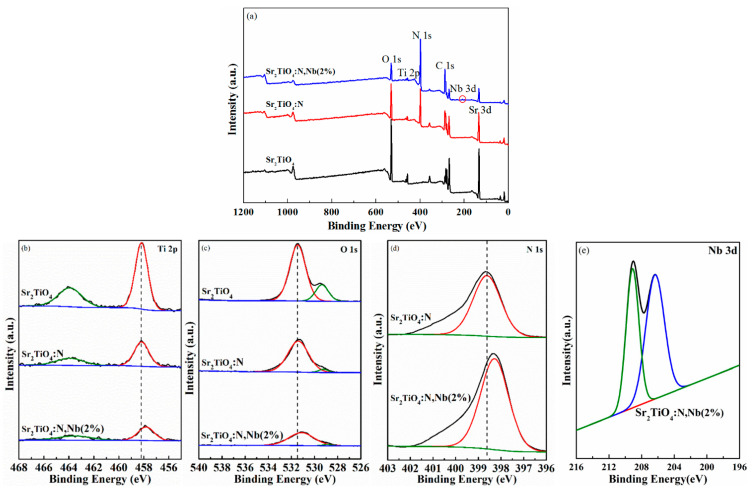
XPS spectra of the pristine and doped Sr_2_TiO_4_: (**a**) full survey spectra, the peak circled in red belongs to Nb 3d; (**b**) Ti 2p; (**c**) O 1s; (**d**) N 1s; (**e**) Nb 3d.

The chemical state and chemical composition on the surfaces of the pristine and doped Sr_2_TiO_4_ were investigated by XPS. The complete measurement scan in Figure 5a showed the presence of Sr, Ti, O, N (except pristine Sr_2_TiO_4_), and Nb (only for Sr_2_TiO_4_:N,Nb(2%)). Figure 5b shows the fine spectra of Ti. The presence of Ti^4+^ was validated by peaks at 458 and 464 eV, which were attributable to Ti 2p_3/2_ and Ti 2p_1/2_ [15,20]. The Ti 2p signal was gradually weakened by simultaneous Nb/N doping, confirming the substitution of Ti with Nb. A similar phenomenon was also observed in La/Fe co-doped Sr_2_TiO_4_ [12]. The peaks located around 529 eV and 531 eV belonged to the lattice oxygen and surface OH^−^ groups [31], respectively (Figure 5c). The signals of lattice oxygen gradually diminished along with N doping, meaning the substitution of lattice oxygen with N^3^^−^ for Sr_2_TiO_4_:N and Sr_2_TiO_4_:N,Nb(2%), which was consistent with XRD results(Figure 1). Due to the strong signal of surface OH^−^, it can be inferred that all samples are hydrophilic [13]. The peak around 398 eV was assigned to lattice N^3−^ as shown in Figure 5d. Along with Nb/N co-doping, this signal of N 1s was enhanced, which demonstrated the introduction of Nb promoting N doping. The 3d_5/2_ and 3d_5/2_ of Nb 3d electrons were detected at binding energies of 207 eV and 209 eV [32], indicating the existence of Nb^5+^. It was worth noticing that binding-energy shifted towards lower binding energy after Nb/N co-doping, which suggests Nb^5+^ doping based on N^3−^ doping was hole-doping. A similar shift was observed in SrCuO_2_ [33].

**Figure 6:**
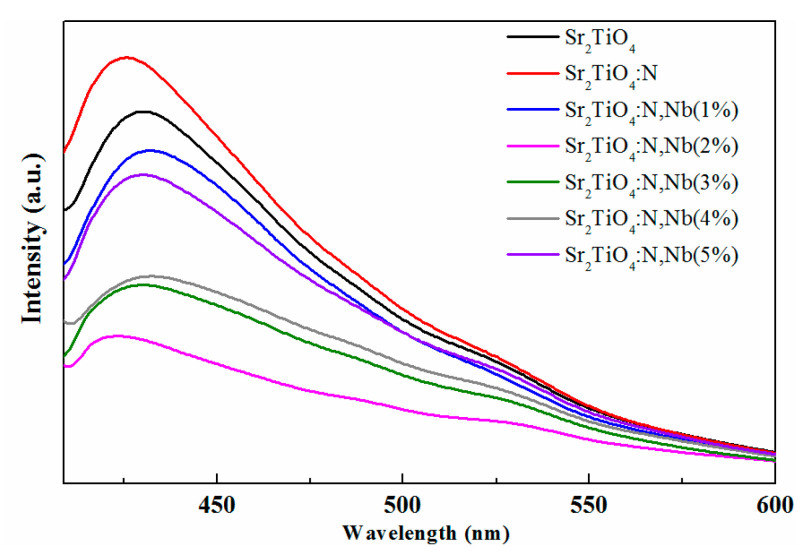
Photoluminescence (PL) spectra of Sr_2_TiO_4_ and Sr_2_TiO_4_:N,Nb.

The charge carrier behaviors of the pristine Sr_2_TiO_4_ and Sr_2_TiO_4_:N,Nb with different Nb-doping content were examined by PL with an excitation wavelength of 325 nm at room temperature. As shown in Figure 6, the emission intensity of Nb/N co-doped Sr_2_TiO_4_ with different Nb doping content were lower than that of pristine Sr_2_TiO_4_, implying that the charge separation capability was enhanced significantly, especially for Sr_2_TiO_4_:N,Nb(2%). The negative charge (O vacancy) introduced by the unequal replacement of N^3−^ and O^2−^ can be balanced by Nb-doping. Therefore, Nb/N co-doping Sr_2_TiO_4_ can reduce the photoelectron-hole recombination probability, which was beneficial to the photocatalytic reaction. Nb^5+^, as a charge balancing agent, can compensate for a charge imbalance caused by N substitution of O, ${\mathrm{Nb}}_{\mathrm{Ti}}^{•}+N_{O}^{\prime }$, just as La played a charge balancing role in La/N co-doped Sr_2_TiO_4_ [13], ${\mathrm{La}}_{\mathrm{Sr}}^{•}+N_{O}^{\prime }$.

### 2.2. Photoelectrochemical Properties of Sr_2_TiO_4_:N,Nb

**Figure 7:**
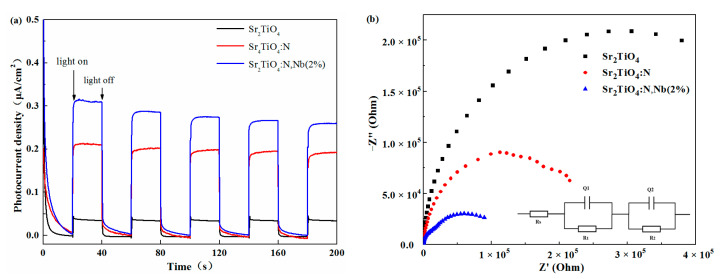
Photocurrent response (**a**) and EIS diagram (**b**) of Sr_2_TiO_4_, Sr_2_TiO_4_:N, and Sr_2_TiO_4_:N,Nb(2%), inset shows the equivalent circuit.

To further confirm the effect of Nb doping on photo-carriers separation and migration of Sr_2_TiO_4_:N,Nb(2%), photochemical measurements were performed. As shown in Figure 7a, the transient-state photocurrent densities of Sr_2_TiO_4_, Sr_2_TiO_4_:N, and Sr_2_TiO_4_:N,Nb(2%) remains stable under chopped light conditions for AM 1.5 illumination. An anodic photocurrent is clearly seen for all investigated samples, indicating that they are n-type semiconductors. The photocurrent density of Sr_2_TiO_4_, Sr_2_TiO_4_:N, and Sr_2_TiO_4_:N,Nb(2%) are 0.034 μA·cm^−2^, 0.199 μA·cm^−2^, and 0.274·cm^−2^ at 1.23 *V*_RHE_, respectively. The photocurrent density of Sr_2_TiO_4_:N,Nb(2%) was 8 times that of Sr_2_TiO_4_ and 1.37 times that of Sr_2_TiO_4_:N, implying the charge-separated significantly more efficiently by the introduction of Nb^5+^ based on N^3−^.

Figure 7b shows the electrochemical impedance spectroscopy (EIS) of Sr_2_TiO_4_, Sr_2_TiO_4_:N, and Sr_2_TiO_4_:N,Nb(2%) and the equivalent circuit, which can further verify the charge transport and transfer. It is well known that the smaller resistance radius indicates the higher separation and migration of electrons and holes. Compared with Sr_2_TiO_4_, Sr_2_TiO_4_:N, and Sr_2_TiO_4_:N,Nb(2%) had a smaller resistance radius, indicating more efficient separation of photocarriers. The result was consistent with the PL spectra and transient photocurrent response. The enhancement of the photocurrent density was attributed to the sufficient light absorption and effective photocarriers separation by introducing Nb^5+^ based on N^3−^ doping.

**Figure 8:**
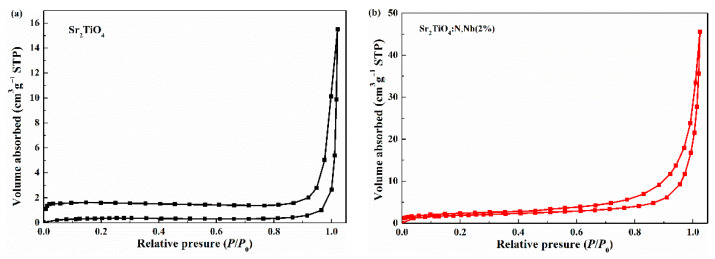
N_2_ adsorption/desorption isotherms Sr_2_TiO_4_ (**a**) and Sr_2_TiO_4_:N,Nb(2%) (**b**).

The specific surface area was tested by an N_2_ adsorption-desorption isotherm at 77 K. As shown in Figure 8, both Sr_2_TiO_4_ and Sr_2_TiO_4_:N,Nb(2%) had typical IV isotherms and H3 hysteresis loops. H3 type hysteresis loops can be observed virtually on adsorbents with a lamellar structure. At high specific pressure, the curves showed an obvious hysteresis loop, which indicated that there was a mesoporous structure in the samples. The average pore diameter was determined using the Barrett–Joyner–Halenda (BJH) methods. BJT desorption average pore diameter (4 V/A) of Sr_2_TiO_4_ and Sr_2_TiO_4_:N,Nb(2%) were 38.24 nm and 23.22 nm, respectively. The specific surface areas of Sr_2_TiO_4_ and Sr_2_TiO_4_:N,Nb(2%) were 1.213 m^2^/g and 6.718 m^2^/g, respectively, which were very low. Specific surface area was not the main reason for the improvement of the photocatalytic degradation performance of Sr_2_TiO_4_:N,Nb(2%).

### 2.3. Photocatalytic Degradation of TC

**Figure 9:**
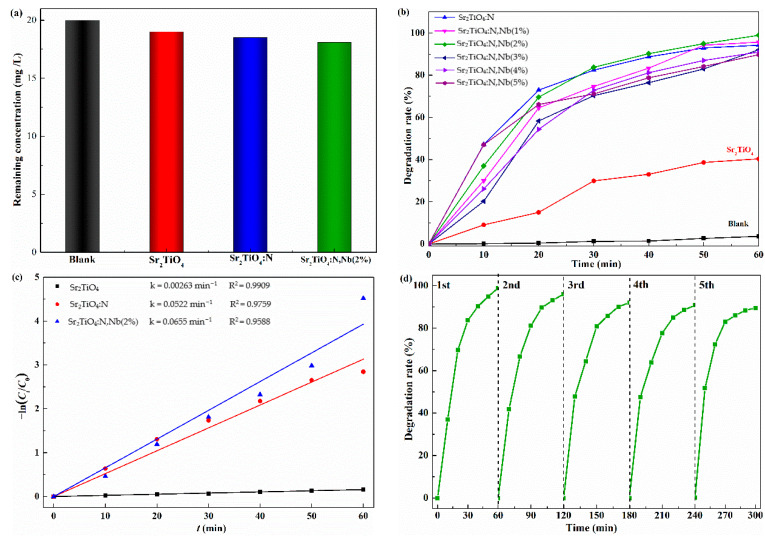
(**a**) Adsorption of different photocatalysts towards TC; (**b**) Self-degradation rate and degradation rate with different photocatalysts of tetracycline under visible light; (**c**) pseudo-first order kinetics curves of the photocatalytic degradation of TC; (**d**) degradation rate of TC after repeated cycles by Sr_2_TiO_4_:N,Nb(2%).

As shown in Figure 9a, the adsorption capacity of photocatalysts towards TC after stirring for 30 min in the dark shows that Sr_2_TiO_4_:N,Nb(2%) exhibited the best adsorption property among them. However, the adsorption capacity was low, which was consistent with BET results. The photocatalytic properties of Sr_2_TiO_4_, Sr_2_TiO_4_:N, and Sr_2_TiO_4_:N,Nb(2%) were investigated by photocatalytic degradation of TC with a 1 g·L^−1^ photocatalyst under visible light. A blank experiment without a photocatalyst was also carried out to exclude the impact of photolysis. The self-degradation rate was 3.6% after 60 min due to negligible photooxygenation reactions in the presence of dissolved oxygen (Figure 9b) [34,35]. As shown in Figure 9b, the photocatalytic performance of all Nb/N co-doping Sr_2_TiO_4_ exhibited a higher degradation rate than prime Sr_2_TiO_4_ under the same conditions. The photocatalytic degradation rate of Sr_2_TiO_4_:N,Nb increased as Nb-doping content increased from 0% to 2%. When the content of Nb exceeded 2%, the degradation rate of Sr_2_TiO_4_:N,Nb decreased, which may be due to the introduction of new defects caused by excessive Nb, ig. ${\mathrm{Nb}}_{\mathrm{Ti}}^{•}+{\mathrm{Ti}}_{\mathrm{Ti}}^{\prime }$. Sr_2_TiO_4_:N,Nb(2%) exhibited the best photocatalytic degradation rate (99%), which was higher than that of prime Sr_2_TiO_4_ (40%) and Sr_2_TiO_4_:N (94%). On the one hand, Nb/N co-doping improved the light absorption in the visible-light region. On the other hand, the recombination probability of Sr_2_TiO_4_:N,Nb decreased, especially Sr_2_TiO_4_:N,Nb(2%). For comparison, the degradation rates of Sr_2_TiO_4_ towards different organic pollutants are shown in Table 3. To the authors’ knowledge, the photocatalytic degradation performance of N-doped Sr_2_TiO_4_ and Nb/N co-doped Sr_2_TiO_4_ has not been reported.

The photocatalytic degradation and fitting results of the TC kinetics followed pseudo-first-order kinetics, as shown in Figure 9c. Sr_2_TiO_4_:N,Nb(2%) showed a maximum apparent reaction rate constant of 0.0655 min^−1^ which was nearly 24.9 times that of Sr_2_TiO_4_ (0.00263 min^−1^) and just 1.25 times that of Sr_2_TiO_4_:N (0.0522 min^−1^). This indicated that N doping had the main effect whereas adding Nb yielded only a slight increase to photocatalytic degradation rates. The maximum apparent reaction rate constant obtained by Sr_2_TiO_4_:N,Nb(2%) was attributed to an appropriate Nb/N co-doping concentration.

To examine the stability and reusability of Sr_2_TiO_4_:N,Nb(2%), five cycles of photodegradation experiments were carried out. After each cycle, the photocatalyst was centrifuged (at 9000 rpm) and washed several times with deionized water for several times and re-placed in a deionized water solution containing fresh TC. As shown in Figure 9d, the degradation rate of Sr_2_TiO_4_:N,Nb(2%) from 99% to 90% after five repeated cycles without an obvious decrease, indicating that Sr_2_TiO_4_:N,Nb(2%) exhibited relatively good stability and repeatability. The decrease of degradation performance could be ascribed to the mass loss in the centrifugation and washing process. Sr_2_TiO_4_:N,Nb(2%) shows good potential in photocatalytic degradation of antibiotic pollutants.

**Figure 10:**
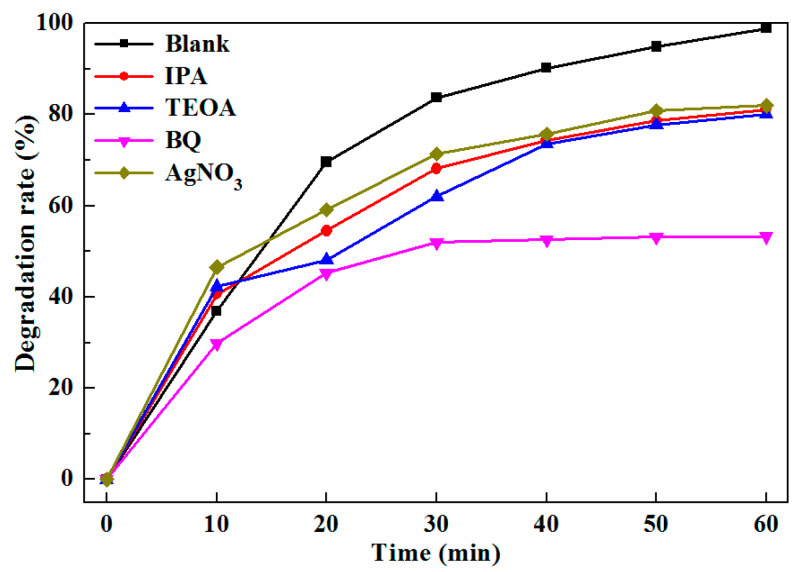
Photocatalytic degradation rate of TC for Sr_2_TiO_4_:N,Nb(2%) in the presence of different scavengers.

To further understand the key active species in the photocatalytic process of TC degradation, active species trapping experiments were performed, as shown in Figure 10. Isopropyl alcohol (IPA, 0.01 M), triethanolamine (TEOA, 0.01 M), p-benzoquinone (BQ, 0.005 M), and AgNO_3_ (0.01M) were added as scavengers for hydroxyl radical (⋅OH), hole (h^+^), superoxide radical ($\cdot O_{2}^{-}$), and photogenerated electrons (e^−^) to TC solution at the presence of Sr_2_TiO_4_:N,Nb(2%), respectively. When the IPA, TEOA, and AgNO_3_ was added, the photodegradation rate of TC only slightly decreased. However, when BQ was added, the degradation rate of TC reduced greatly, indicating that superoxide radical ($\cdot O_{2}^{-}$) was the main active species during the photocatalytic degradation of TC.

### 2.4. Improvement Mechanism of Photocatalytic Performance

**Figure 11:**
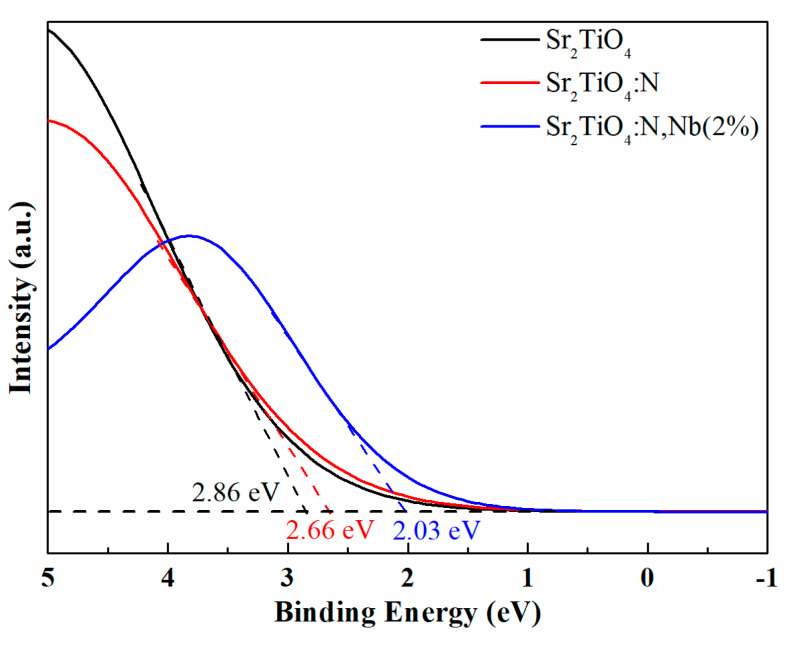
XPS valence band spectra of Sr_2_TiO_4_, Sr_2_TiO_4_:N, and Sr_2_TiO_4_:N,Nb(2%).

The effect of N doping and Nb/N co-doping on the valence bands of Sr_2_TiO_4_ was evaluated by XPS valence band (VB) spectra. As shown in Figure 11, valence band potential (*E*_VB_, _XPS_) measured by XPS valence band spectra of Sr_2_TiO_4_, Sr_2_TiO_4_:N, and Sr_2_TiO_4_:N,Nb(2%) were 2.86, 2.66, and 2.03 eV, respectively. *E*_VB_ vs. SHE can be deduced according to the formula: *E*_VB, NHE_ = *φ* + *E*_VB, XPS_ − 4.5 (*φ* is the work function of the instrument: 4.59 eV, 4.5 eV vs. vacuum level is 0 V vs. SHE) [39,40,41,42]. Thus, *E*_VB, NHE_ of Sr_2_TiO_4_, Sr_2_TiO_4_:N, and Sr_2_TiO_4_:N,Nb(2%) were calculated to be 2.95 eV, 2.75 eV, and 2.12 eV, respectively. The conduction band (VB) minimum of Sr_2_TiO_4_, Sr_2_TiO_4_:N, and Sr_2_TiO_4_:N,Nb(2%) are calculated to be −0.53 eV, −0.27 eV, and −0.83 eV(vs. SHE) according to these valance band and bandgap values (Figure 4b) mentioned above. The conduction band minimum of Sr_2_TiO_4_ reported in the previous literature varies widely and ranges from −0.254 eV to −0.87 eV [16,17,20,41], and the conduction band minimum of N-doped Sr_2_TiO_4_ and Nb/N co-doped Sr_2_TiO_4_ have not been reported.

**Figure 12:**
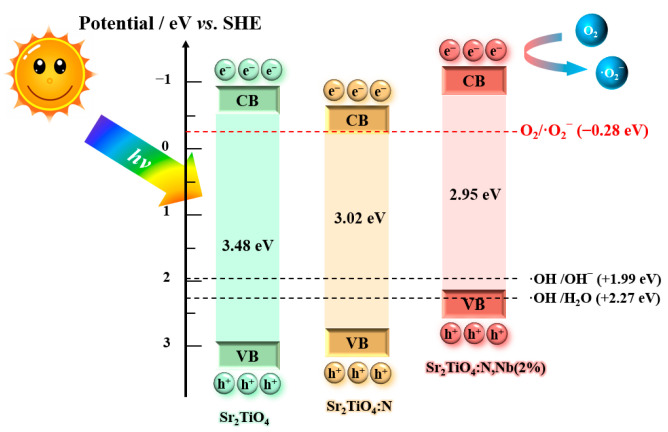
Schematic band structures of Sr_2_TiO_4_, Sr_2_TiO_4_:N, and Sr_2_TiO_4_:N,Nb(2%). The potential of $O_{2}/▪O_{2}^{-}$, $\mathrm{OH}/{\mathrm{OH}}^{-}$, and $\mathrm{OH}/H_{2}O$ are shown in the diagram.

According to our experimental results of XPS valence band spectra (Figure 11) and UV-Vis spectra (Figure 4b), schematic band structures of Sr_2_TiO_4_, Sr_2_TiO_4_:N, and Sr_2_TiO_4_:N,Nb(2%) were illustrated in Figure 12. The pure Sr_2_TiO_4_ had a wide bandgap. In contrast, Sr_2_TiO_4_:N displayed visible light response derived from N orbital. The state of the oxygen vacancies (accompanied by N-doping) was located below the conduction band minimum, which was consistent with previous literature about SrTiO_3_ co-doped with N and La [21]. Regarding Sr_2_TiO_4_:N,Nb(2%), Nb/N co-doping uplifted the valence band furthermore. As we know, the more negative the valance band, the higher reduction ability of photo-generated electrons to generate superoxide radicals ($▪O_{2}^{-}$). The conduction band potential of Sr_2_TiO_4_:N,Nb(2%) was more negative than Sr_2_TiO_4_, Sr_2_TiO_4_:N, and $O_{2}/▪O_{2}^{-}$ (−0.28 eV), so electrons can more easily transfer to the oxygen molecules, producing $▪O_{2}^{-}$. The uplift of conduction band after cationic doping had also been observed in Ag doping Sr_2_TiO_4_ [16].

Hydroxyl radical (OH) may be difficult to produce by the photogenerated holes, directly, because the valence band position (+2.12 eV) in Sr_2_TiO_4_:N,Nb(2%) was higher than that of $\mathrm{OH}/H_{2}O$ (+2.27 eV) and slightly more positive than that of $\mathrm{OH}/{\mathrm{OH}}^{-}$(1.99 eV). Therefore, $▪O_{2}^{-}$ is the predominant active species in the degradation of TC, which coincided with the previous result (Figure 10). A similar situation was also discovered in Bi_2_WO_6_ [43,44].

## 3. Materials and Methods

### 3.1. Chemicals

The chemicals in this work were obtained from Shanghai Maclin Biochemical Technology Co., Ltd. (Shanghai, China) and Tianjin Yongda Chemical Reagent Co., Ltd. (Tianjin, China), without any further purification.

### 3.2. Materials Synthesis

#### 3.2.1. Synthesis of Sr_2_TiO_4_

Firstly, 0.01 mol tetrabutyl titanate was dissolved in 50 mL of absolute ethanol, and 0.07 mol citric acid was injected as a complex agent (solution A). Secondly, 0.02 mol Sr(NO_3_)_2_ was dissolved in deionized water (solution B). Solution B was added dropwise to solution A to gain a transparent mixed solution. The mixed solution was heated and stirred in a water bath at 60 °C to form a homogeneous transparent sol. After aging for 24 h, the sol was dried at 120 °C for several hours to form a dry gel. Finally, the obtained gel was ground and calcined at 1000 °C for 4 h (heating rate 10 °C·min^−1^) to obtain Sr_2_TiO_4_ powder.

#### 3.2.2. Synthesis of N-Doped Sr_2_TiO_4_

The preparation process of N-doped Sr_2_TiO_4_ was as follows: After mixing urea and Sr_2_TiO_4_ uniformly (m(urea):m(Sr_2_TiO_4_) = 1.5:1), the mixture was calcinated at 400 °C for 2 h, cooled naturally, and grinded. N-doped Sr_2_TiO_4_ was labeled as Sr_2_TiO_4_:N.

#### 3.2.3. Synthesis of Nb/N Co-Doped Sr_2_TiO_4_

The preparation process of Nb/N co-doped Sr_2_TiO_4_ was as follows: Nb-doped Sr_2_TiO_4_ was prepared first, which was the same as that of Sr_2_TiO_4_, except for the addition of NbCl_5_ in solution B (Nb/(Nb + Ti), which was 1 at%, 2 at%, 3 at%, 4 at%, 5 at%. Nb/N co-doped Sr_2_TiO_4_ was prepared the same as that of N-doped Sr_2_TiO_4_, except that urea was mixed with Nb-doped Sr_2_TiO_4_. Nb/N co-doped Sr_2_TiO_4_ with different Nb doping content were denoted as Sr_2_TiO_4_:N,Nb(1%), Sr_2_TiO_4_:N,Nb(2%), Sr_2_TiO_4_:N,Nb(3%), Sr_2_TiO_4_:N,Nb(4%), and Sr_2_TiO_4_:N,Nb(5%), respectively.

### 3.3. Characterization

The crystal structure analysis was carried out by a Japanese D/MAX2500PC X-ray diffractometer employing Cu Kα radiation; the surface morphology was investigated by a Japan JEM-2010 transmission electron microscope (TEM); a specific surface and pore size analyzer (3H-2000PM1, BeiShiDe Instrument Technology Co., Ltd., Beijing, China) was used to calculate the specific surface area (BET) and pore size of the sample; the optical properties were investigated by an Ultraviolet-Visible spectrophotometer (Lambda750, PerkinElmer, Norwalk, CT, USA); the photoluminescence (PL, SR830) with the excitation at 325 nm was characterized using a fluorescence spectrometer; the chemical composition and valence band spectrum were detected by X-ray photoelectron spectroscopy (Thermo Scientific, Waltham, MA, USA, ESCALAB 250XI).

### 3.4. Photoelectrochemical Measurements

Photocurrent density-time (*I*-*t*) and electrochemical impedance spectroscopy (EIS) were characterized on an electrochemical workstation (CHI660D, Shanghai Chenhua Instrument, Shanghai, China) through a three-electrode system. Three-electrode system was set up using 0.5 M Na_2_SO_4_ solution, Ag/AgCl as reference electrode, Pt foil as counter electrode, and photoelectrodes fabricated by these sample powders on FTO glass as working electrodes. During the measurements of photocurrent density, simulated sunlight was irradiated with an intensity of 100 mW·cm^−2^ (AM1.5G filter) under chopped light at a bias of 1.23 V vs. RHE. The EIS was measured with a frequency from 100 kHz to 0.01 Hz with an AC amplitude of 5 mV.

### 3.5. Photocatalytic Degradation of TC

The photocatalytic performances of the samples were assessed by photocatalytic degradation towards TC. In total, 50 mg Sr_2_TiO_4_ or 50 mg Sr_2_TiO_4_:N,Nb was dispersed in 50 mL of TC solution at a concentration of 20 mg·L^−1^. Before irradiation, the mixtures were continuously stirred in the dark for 30 min to reach adsorption-desorption equilibrium. Then, a photocatalytic reaction was performed under visible light using a 5 W LED lamp (white light, λ > 420 nm, 250 mW/cm^2^). Extract 3 mL of TC solution every 10 min and centrifuge at 9000 rpm for 5 min. The TC degradation rate was calculated as follows:

(1) $$\mathrm{Degradation}\;\mathrm{rate}=\frac{C_{0}-C_{t}}{C_{0}}\times 100\%=\frac{A_{0}-A_{t}}{A_{0}}\times 100\%$$

where *C*_0_ (*A*_0_) and *C_t_* (*A_t_*) represent the TC’s concentration (absorbance at 357 nm) of initial and after min irradiation, respectively.

In order to ascertain the active species during the photocatalytic degradation process, the active species capture experiment was carried out, similar to the photocatalytic degradation of TC experiments, except that TC solution was replaced by TC and scavenger.

## 4. Conclusions

In summary, Nb/N co-doped layered perovskite Sr_2_TiO_4_ (Sr_2_TiO_4_:N,Nb) with varying percentages (0–5 at%) of Nb were successfully synthesized by sol-gel and calcination. Nb/N could co-dope into Sr_2_TiO_4_ with a slight unit cell expansion, which was confirmed by X-ray diffraction. Nb/N co-doped Sr_2_TiO_4_ showed better photocatalytic performance for tetracycline photocatalytic degradation than pristine Sr_2_TiO_4_ under visible light, especially Sr_2_TiO_4_:N,Nb(2%). Sr_2_TiO_4_:N,Nb(2%) showed optimal photocatalytic performance with the 99% degradation after 60 min visible light irradiation, which was higher than pristine Sr_2_TiO_4_ (40%). Nb/N co-doping enhanced the photocatalytic activity by broadening light response to the visible region and reducing the photogenerated carrier recombination. Nb/N co-doping had a slight effect on morphology and the average grain size of Sr_2_TiO_4_. Sr_2_TiO_4_:N,Nb(2%) showed good stability and recyclability after five cycles photocatalytic degradation reaction. The superoxide radical ($\cdot O_{2}^{-}$) was the leading contributor to tetracycline degradation. Nb/N co-doping strategy in this work affords insight to realizing visible-light-driven perovskite-type semiconductors for wastewater treatment.

## Figures and Tables

**Table 1:** Properties of Sr_2_TiO_4_ doping with differentiation.

Doping Elements	A-Site/B-Site or O-Site Doping	Doping Content	*E*_g_ (eV)	References
Cr	B	5 at%	~1.5	[15]
Ag	A	2.5 at%	3.05	[16]
F	O	3 at%	3.20	[17]
Chalcogens (S, Se, Te)	O	--	0~2.99	[18]
La/N	A/O	10 at%/-	2.2	[13]
Cr/F	B/O	5 at%/40 at %	~2.5	[19]
La/Rh	A/B	1.5 at%/3 at %	2.43	[20]
La/Fe	A/B	1.5 at%/3 at %	~2.75	[12]

**Table 2:** Average crystallite size of Sr_2_TiO_4_, Sr_2_TiO_4_:N, and Sr_2_TiO_4_:N,Nb.

	Sr_2_TiO_4_	Sr_2_TiO_4_:N	Sr_2_TiO_4_:N,Nb(1%)	Sr_2_TiO_4_:N,Nb(2%)	Sr_2_TiO_4_:N,Nb(3%)	Sr_2_TiO_4_:N,Nb(4%)	Sr_2_TiO_4_:N,Nb(5%)
Average crystallite size	43 nm	65 nm	64 nm	55 nm	68 nm	71 nm	61 nm

**Table 3:** Photocatalytic properties of Sr_2_TiO_4_.

Samples	Doping Elements	Organic Pollutants	Degradation Rates	Irradiation System	References
Sr_2_TiO_4_	——	methylene orange	32% (120 min)	UV lamp (400 W Osram lamps)	[36]
Sr_2_TiO_4_	——	methylene orange	3.2% (180 min)	Xenon lamp (300 W without filter)	[37]
Sr_2_TiO_4_	——	methylene orange	78% (120 min)	UV lamp (λ = 253.7 nm, 2200 mW·cm^−2^)	[38]
Sr_2_TiO_4_	La	methylene orange	90.5% (120 min)	UV lamp (λ = 253.7 nm, 2200 mW·cm^−2^)	[38]
Sr_2_TiO_4_	——	tetracycline	40% (60 min)	LED lamp (λ > 420 nm)	This work
Sr_2_TiO_4_	Nb/N	tetracycline	99% (60 min)	LED lamp (λ > 420 nm)	This work

## Data Availability

Not applicable.
